# A system-level, molecular evolutionary analysis of mammalian phototransduction

**DOI:** 10.1186/1471-2148-13-52

**Published:** 2013-02-23

**Authors:** Brandon M Invergo, Ludovica Montanucci, Hafid Laayouni, Jaume Bertranpetit

**Affiliations:** 1 Institute of Evolutionary Biology (CSIC-Universitat Pompeu Fabra), CEXS-UPF-PRBB, Barcelona, Catalonia, Spain

## Abstract

**Background:**

Visual perception is initiated in the photoreceptor cells of the retina via the phototransduction system. This system has shown marked evolution during mammalian divergence in such complex attributes as activation time and recovery time. We have performed a molecular evolutionary analysis of proteins involved in mammalian phototransduction in order to unravel how the action of natural selection has been distributed throughout the system to evolve such traits.

**Results:**

We found selective pressures to be non-randomly distributed according to both a simple protein classification scheme and a protein-interaction network representation of the signaling pathway. Proteins which are topologically central in the signaling pathway, such as the G proteins, as well as retinoid cycle chaperones and proteins involved in photoreceptor cell-type determination, were found to be more constrained in their evolution. Proteins peripheral to the pathway, such as ion channels and exchangers, as well as the retinoid cycle enzymes, have experienced a relaxation of selective pressures. Furthermore, signals of positive selection were detected in two genes: the short-wave (blue) opsin (*OPN1SW*) in hominids and the rod-specific *Na*^+^/ *Ca*^2+^, *K*^+^ ion exchanger (*SLC24A1*) in rodents.

**Conclusions:**

The functions of the proteins involved in phototransduction and the topology of the interactions between them have imposed non-random constraints on their evolution. Thus, in shaping or conserving system-level phototransduction traits, natural selection has targeted the underlying proteins in a concerted manner.

## Background

Visual perception begins with phototransduction, the process by which a light stimulus is converted into a neuronal signal. While the structures of phototransduction systems have diverged between metazoan phyla, the underlying principles remain the same: photons of light are absorbed by light-sensitive chromophores bound to opsin proteins present in photoreceptor cells. The opsins trigger a prototypical G-protein-coupled receptor-mediated signaling cascade that amplifies the opsin response and results in the hyperpolarization of the cell. This initiates a neuronal signal that is transmitted to the visual cortex in the brain for processing. The cell must then efficiently return to an inactive state through the deactivation of the opsin, the recycling of the chromophore in the canonical retinoid cycle and the re-opening of the cGMP-gated ion channel in order to be able to respond to further stimuli (for an in-depth review of the process, see [1]). The phototransduction system of mammals has been the focus of much research. The rod system, which is responsible for scotopic, or low-light vision, has been intensely studied in a wide range of species while the cone system, which is responsible for photopic, or high-light vision, is less well-understood. However recent years have seen a large advance in our knowledge of the specifics of cone phototransduction.

Accurate visual perception of the environment carries clear evolutionary advantages. A well-known evolutionary development in mammalian phototransduction is in the evolution of the cone opsin repertoire. Early mammals are thought to have gone through a nocturnal phase, during which genes from two families of opsins were lost, leaving them with only two remaining cone opsins and without color vision [2]. Subsequently, due to a gene duplication event during their divergence, Old World monkeys acquired a third cone opsin gene and have regained color vision [3]. In addition to the evolution of the cone opsin repertoire, the molecular evolution of individual opsin proteins has been well-characterized in mammals [4-13], as has their phylogenetic relationship to other extant opsin genes [14]. Further differences between mammalian species are known to exist in the spatial patterning of photoreceptor cells on the retina. For example, while the three cone types of humans are distributed in a stochastic mosaic pattern of varying concentrations throughout their retinas, the short-wave and medium-wave opsins of mice are expressed in opposing gradients along the superior (dorsal/ventral) axis [15]. Finally, primates have developed the fovea, a highly concentrated region composed exclusively of cone cells in the center of the retina, which imparts very high acuity.

While much attention has been given to the evolution of opsin proteins, less has been afforded to the molecular evolution of the phototransduction system as a whole throughout mammalian divergence (but see [16] and [17]). It is clear that variation exists in the rates of characteristics of photoresponses measured in different mammalian species, such as in response activation or recovery time. Some variability in response characteristics may be due to, for example, photoreceptor cell volume or protein expression levels (see [18] for a comprehensive review of the molecular dynamics of phototransduction). However, it is, at the same time, expected that molecular evolution of the proteins involved in phototransduction has contributed to this response variation. In fact, it is possible to recognize in extant gene regions the footprint of past selective events, be they purifying selection or positive (adaptive) selection.

When considering the evolution of a trait such as the activation or recovery times of this signaling process, one must first recognize the traits as being of a complex nature: the time required for the cell to reach an activated state is not the result of the action of a single protein but depends instead upon many proteins and a complex set of interactions between them. A traditional evolutionary biology approach to studying the differences between species of this system might consider the phenotypic evolution of the system-level trait as a whole, while a molecular evolutionary approach might typically focus on how key individual proteins have evolved over species divergence. A more recent method is to encode the molecular interactions comprising the system as a network and to investigate how the evolutionary histories of the molecules relate to the topological characteristics of the system [19-28]. A network representation of a system provides a convenient mathematical abstraction that captures some of the functional characteristics of the underlying molecules. Typically, the proteins are represented as the nodes of the network and the edges connecting the nodes represent some form of interaction between the molecules, such as direct protein-protein interactions, shared metabolites or regulatory relationships. Topological measurements of the nodes may then be measured, such as their centrality or their connectivity. These measurements may be interpreted to reflect the importance and the role of a protein in the system: a highly connected node, representing a protein with many interactions, may be a critical component of the system, for example.

Many methods to estimate rates of molecular evolution during species divergence have been proposed. Some of the most commonly used methods are those that are implemented in the Phylogenetic Analysis by Maximum Likelihood (*PAML*) software package [29], particularly the codon-based analyses of the program codeml. Several models of codon evolution are implemented, with more complex and realistic models expanding upon the simpler ones, allowing tests by likelihood-ratio of hypotheses such as site-specific events of positive selection. When considering the evolution of a collection of genes, one must consider the sacrifice in model power made to gain sensitivity by adding more parameters; in order to accurately estimate evolutionary rates with such models, larger datasets, consisting of more species of larger divergence, are necessary. This is important when considering molecular evolution within a system: when deeper phylogenies are considered, the likelihood of fundamental structural differences in the system between species increases, due to gain or loss of genes or protein-protein interactions, impacting one’s ability to investigate system-level, evolutionary constraints acting on a molecule. Indeed, at a deep enough phylogeny, we find very different phototransduction cascades altogether, resulting from relatively few ancient opsin mutations [30]. For these reasons, while simpler models of codon evolution may have less realistic assumptions, their use on relatively shallow phylogenies provides more robust rate estimates for comparing the evolutionary histories of proteins interacting in a system.

The studies performed thus far investigating how pathway structure restrains molecular evolution have shown that there exists a clear correlation between pathway topology and evolutionary rates of the system’s proteins, however the specific relationship varies from pathway to pathway. In order to determine how the phototransduction system has evolved during mammalian divergence, we have performed a molecular evolutionary analysis of a comprehensive list of genes that encode proteins involved in the process across a range of mammalian species, following the strategy of Montanucci *et al.*[19]. These proteins belong to the phototransduction signaling cascade, the canonical retinoid cycle and the developmental process which commits a photoreceptor progenitor cell to its ultimate fate as a rod or a cone. In addition to testing for evidence of positive selection events, we also compared estimated rates of evolution of the genes encoding the proteins to infer relative strengths of purifying selection acting on them. We then ascertained the distribution of the selective pressures throughout the system, by working with two characterizations of it: a simple protein classification system and a network encoding of the proteins’ interactions. This approach has allowed us to determine that selection has been non-randomly distributed throughout this system, with some cases of positive selection and and with an uneven distribution of the strength of purifying selection, thus providing evidence for how natural selection on the system as a whole has shaped the evolution of the underlying proteins for its adaptation.

## Results and discussion

### System-wide distribution of selective pressures

In total, 57 genes were identified as being directly relevant to the phototransduction system in mammals, including genes encoding proteins of the signaling cascade, the retinoid cycle, and key proteins involved in the final steps of photoreceptor cell-type (rod versus cone) determination. Of these, three were omitted from the analysis due to a lack of functional orthologs in some species: *GRK7* (G-coupled protein receptor kinase 7; not present in the mouse or rat genomes), *GUCA1C* (guanylate cyclase activator 1C; not present in the mouse or rat genomes) and *OPN1LW* (long-wave/red cone opsin; only present in Old World primate genomes).

**Figure 1:**
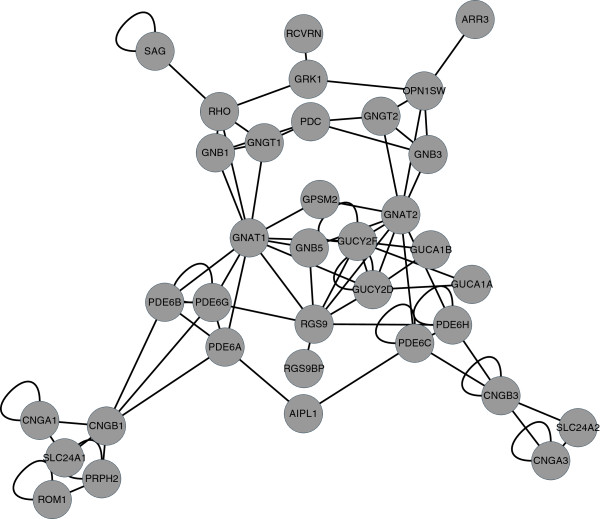
**Phototransduction network.** A network representation of the phototransduction signaling cascade. Nodes represent proteins and edges represent physical interactions between them (Additional file 1: Table S1).

In this research, we have applied two methods of characterizing the phototransduction system in order to perform system-level analyses. In the first, we categorized the proteins involved in the process to determine if different groups of proteins evolved at significantly different rates than others. We chose three systems of categorization: photoreceptor cell-type specificity (rod- or cone-specific or shared), process specificity (phototransduction, retinoid cycle, or developmental photoreceptor cell-type determination), and a functional classification of each protein (opsin, G protein, etc.) (Additional file 1: Table S1). The second representation of the system which we chose to implement is that of a network of the protein-protein interactions which occur during the phototransduction signaling cascade (Figure 1). For this, all known interactions involved in the system were carefully curated from research literature to arrive at a network of high confidence (Additional file 1: Table S2). This is important, as there tend to be inaccuracies and omissions in many pathway representations found in public databases, which may introduce too many errors for non-interactome-scale analyses [31,32].

The strength of purifying selection that has acted on each gene was estimated according to the ratio, *ω*, of the rate of non-synonymous substitution (*d*_*N*_) to the rate of synonymous substitution (*d*_*S*_) over the entire length of the gene (Additional file 1: Table S3). Single rates were calculated across the entire phylogenetic tree, which represented the relationships between nine mammalian species with high-quality genome data: human, chimpanzee, gorilla, orangutan, macaque, marmoset, rat, mouse and dog. As is usually expected, *ω* for every gene was less than one. Since synonymous substitutions can generally be assumed to be of little adaptive significance compared to non-synonymous substitutions, the typical interpretation of *ω* values is of an inverse relationship with the strength of purifying selection, so that lower *ω* values indicate stronger purifying selection. However, rates of synonymous substitution may be weakly affected by translation-related processes, which may manifest, for example, in a relationship between evolutionary rates and protein length [33]. We checked for such a relationship between *ω* and the length of the proteins encoded by the human genes but found none.

**Figure 2:**
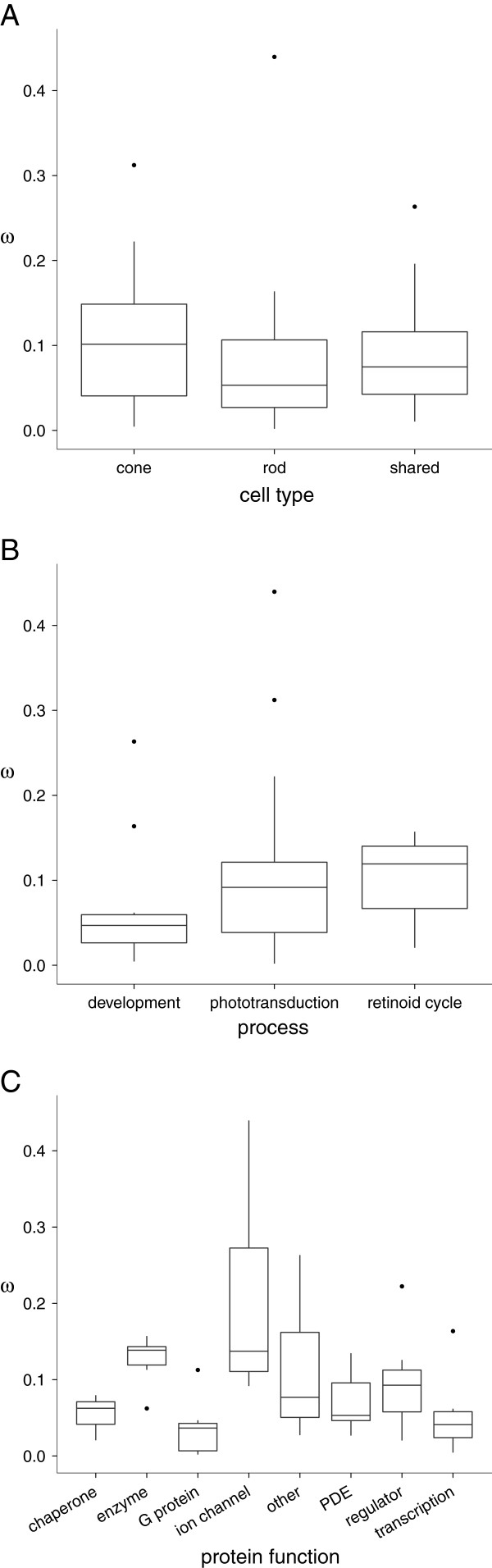
**Distribution of selective pressures by classification.** Boxplots comparing the distributions of *ω* values according to different gene-classification schemes. **A**: The photoreceptor cell-type specificity of the encoded proteins. **B**: The specific process in which the encoded protein is primarily active. **C**: The specific function of the protein (note that here “enzyme” refers to the enzymes of the retinoid cycle).

Given the recent evolution of trichromacy in Old World monkeys, one might hypothesize that the entire cone phototransduction system would have then experienced subsequent evolution, re-tuning to a newly available visual environment. This was found not to be the case. While the photoreceptor cell-type specificity of the proteins was found to affect rates of synonymous substitution (*χ*^2^=8.27, *df*=2, *p*=0.016), with cone-specific genes having lower rates, non-synonymous substitution rates and *ω* were unaffected (Figure 2A). Thus, there is no significant difference in selective pressures acting on the two phototransduction systems. The process to which proteins belong in this system (development, phototransduction, or the retinoid cycle) significantly affected the rates of non-synonymous substitution (*χ*^2^=8.57, *df*=2, *p*=0.014). The relationship between process and *ω*, however, was not strong enough to be considered significant (Figure 2B; *χ*^2^=5.82, *df*=2, *p*=0.054).

The two previous classifications were used to investigate relationships between selective pressures experienced by closely related, but distinct, processes, namely the cone and rod phototransduction systems. Clearly, these processes as wholes do not have a history of significantly different selective pressures. Classifying the proteins according to their function, on the other hand, allows for patterns both between and within the processes to be probed. Indeed, this classification scheme revealed a significant source of variation in rates of non-synonymous substitution (*χ*^2^=28.3, *df*=7, *p*<0.001) and *ω* (Figure 2C; *ω*: *χ*^2^=24.0, *df*=7, *p*=0.001). In this case, we see that the chaperon proteins of the retinoid cycle, the G proteins of the phototransduction signaling cascade and the transcriptional regulation proteins of the developmental process have all been highly constrained in their evolution. The enzymes of the retinoid cycle and the ion channels/exchangers of the phototransduction cascade, on the other hand, have experienced much more relaxed purifying selection, indicating that there has been relatively more evolution in these proteins over the diversification of mammals.

**Figure 3:**
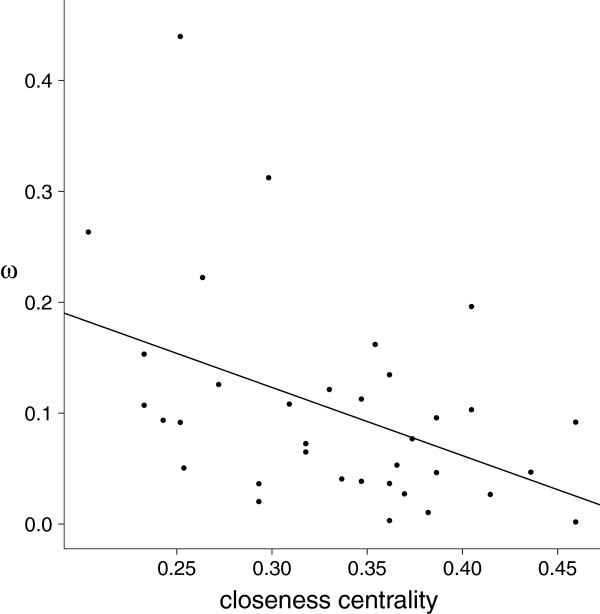
**Distribution of selective pressures by network centrality. ***ω* values compared to the closeness centralities of proteins in the network representation of the phototransduction signaling pathway. A least-squares regression line is shown (*ρ*=−0.4202, *p*=0.012).

By taking a different approach and reducing the complexity of the problem by building a network representation of the phototransduction signaling process, we also found non-random patterns in the distribution of selective pressures within the system. A network representation of the system offers the advantage of encoding the topology of interactions between proteins, allowing for a sense of position and importance within the system. Of the measurements of network importance we calculated, closeness centrality was found to have a significant negative correlation with rates of non-synonymous substitution and with *ω* values (Figure 3; *ω*: *ρ*=−0.4202, *p*=0.012) as a result of a significant correlation with *d*_*N*_ (*ρ*=−0.4766, *p*=0.004) and no relation with *d*_*S*_. This indicates that, indeed, the proteins which are most topologically central to the process of converting a light stimulus to a neuronal signal have been most constrained in their evolution, while those which are on the periphery of the system have experienced increased rates of evolution. Interestingly, if we classify the proteins according to function as we did before and compare the closeness centrality measurements of these classes, we see significant differences. G proteins, for example, have much higher centrality while ion channels/exchangers have much lower centrality. From this we may conclude that both methods of abstraction that we used, functional classification and network representation, retained some of the same relevant information of the system. Finally, we were able to detect a correlation in the selective pressures experienced by interacting proteins in the network, with a weak relationship between non-synonymous substitution rates of interacting genes (*ρ*=0.3404, *p*=0.045). Thus, while a protein’s relative position in the system affects the strength of selection acting on it, it is apparently not strongly affected by constraints acting on its direct interacting partners.

### Tests of positive selection

We found two cases of positive selection events in the system. The first significant signal of positive selection was detected in the short-wave (blue) cone opsin (*OPN1SW*) on the branch of the phylogenetic tree leading to the hominids (uncorrected *p*<0.001; corrected *p*=0.045). The branch-site test of positive selection has low power, typically only correctly identifying less than 20% of positive selection events [34]. After stringent correction for multiple testing, with the assumption of correlated evolution of genes within and across branches, this was the only phototransduction-related gene with a resulting *p*-value less than one on this branch. Thus, an adjusted *p*-value near to the critical value of 0.05 can still be considered to point to an event of positive selection. Furthermore, this gene previously has been found to be under positive selection in primates [35,36].

Two possible sites of positive selection were indicated by posterior tests. The first, amino acid position 289 (*p*(*ω*>1)=0.778), shows a change from an Alanine to a Serine in the phylogenic branch leading to the hominids. This residue is in the seventh transmembrane helix of the protein, near to the retinylidene Schiff’s base formation at position 293. This site has been shown to have an effect on the spectral tuning of some opsin proteins. A replacement of an Alanine by a Serine at this position puts the chromophore’s Schiff-base group in a more polar environment, which results in a blue shift of approximately 10nm in RH1 opsins (Rocha and Danchin 2004; Fasick and Robinson 1998; Lin *et al.* 1998; Janz and Farrens 2001) and 8nm in SWS2 opsins [37]. The reverse replacement leads to a red shift of approximately 8nm in RH1 opsins [38] and 18-28nm in M/LWS opsins [39]. It is somewhat surprising, then, that the human SWS1 gene was shown to have no change in its peak-absorbance wavelength when this residue was experimentally changed from a serine to an alanine [40]. If this position has indeed experienced positive selection in hominids, and it is confirmed that it has no effect on spectral tuning in hominids, then further experimentation must be done in order to determine the functional significance of this residue substitution.

The second possible location of positive selection in this protein was at amino acid position 343 (*p*(*ω*>1)=0.755), which shows a substitution of Threonine for Serine in the branch leading to the hominids. As this residue is located at the C-terminus of the protein, it is a probable site for phosphorylation by rhodopsin kinase (*GRK1*) or by the cone-specific receptor kinase, *GRK7*, in the species which express it. However, as these are Serine/Threonine-specific kinases, a phosphorylation site was neither gained nor lost. It has been shown that, in rhodopsin, only the number and not the identity of phosphorylated sites influences the deactivation of the opsin (Doan *et al.* 2006). Thus, even if there were differences in the kinases’ specificities for Serine versus Threonine, which does not seem to be the case (Francesc Posas, personal communication), it is unlikely to be functionally relevant to the phosphorylation process’s influence on system deactivation. However, it should be noted that the corresponding amino acid residue in rhodopsin, position 343, has been found to be one of two phosphorylation sites which are critical for the efficient binding of arrestin (Zhang *et al.* 1997), and, due to the competitive nature of the binding of arrestin or transducin to activated rhodopsin, the site may also affect transducin binding. Whether a substitution of a threonine at this site might influence the binding of these proteins to the activated opsin, and thus be subject to adaptation, merits further investigation.

Evidence of positive selection was also detected in a second gene, the rod-specific *Na*^+^/ *Ca*^2+^, *K*^+^ ion exchanger (*SLC24A1*) on the phylogenetic branch leading to the rodents (uncorrected *p*<0.001; corrected *p*=0.008). Two amino acids which were potentially the targets of positive selection were at positions 254 (*p*(*ω*>1)=0.843) and 335 (*p*(*ω*>1)=0.894). Both of these positions lie in an extracellular region of the protein near the N-terminus (Reiländer *et al.* 1992). Thus, it is not clear whether these positions are functionally relevant nor why they may have been targeted for positive selection. However, with an *ω* value of 0.44, a clear outlier among the other genes, there may have been much functional evolution throughout this protein.

Recently, rod arrestin (SAG) was found to show evidence of positive selection [35]. We also found a strong signal in this gene, however, upon closer inspection of the alignment it was determined that the region estimated by our tests to be under positive selection was very poorly aligned. Both the chimpanzee and orangutan sequences at nucleotide positions 1023 through 1046 are identical to each other but are only 26% similar to the human and gorilla sequences. The macaque sequence was 30% similar to the human and gorilla sequences and 34% similar to the chimpanzee and orangutan sequences. This region corresponds to the entirety of exon 13 (Ensembl ID: ENSE00001760374) in the human sequence. Thus, we speculated that the orangutan and chimpanzee exons were improperly predicted. By performing a BLAT search using the entire human DNA sequence of the gene, we recovered the proper exon sequences for both the chimpanzee and the orangutan. The proper exon sequence could not be recovered for the macaque, however, so the region was ultimately masked from the alignment. Upon removing the region, no further signals of positive selection were detected in this gene. Thus, the previously published result is likely due to sequencing or annotation inaccuracies.

## Conclusions

We have provided evidence that, during the divergence of primates, natural selection has tuned the performance of the first steps of vision through non-random targeting of member proteins within the system. Positive selection events were detected in the short-wave cone opsin (OPN1SW) in hominids as well as in the rod-specific *Na*^+^/ *Ca*^2+^, *K*^+^ ion exchanger (SLC24A1) in rodents. Proteins which are topologically central in a network representation of the pathway, such as the G proteins, have been under strong purifying selection. Meanwhile, the proteins at the periphery of the network, especially the cyclic nucleotide-gated ion channels and the *Na*^+^/ *Ca*^2+^, *K*^+^ ion exchangers, have experienced relaxed purifying selection. Furthermore, we have found that the enzymes involved in the canonical retinoid cycle have also seen a relative relaxation in the strength of purifying selection. These relaxations in purifying selective pressure could be responsible for some of the species differences in phototransduction response times, via a progressive accumulation of amino acid changes in the proteins. The strong selective constraint imposed on transcriptional regulation proteins active in photoreceptor development indicates that evolved differences between species in retinal patterning of photoreceptor cells, on the other hand, must have largely arisen by other means such as by the evolution of regulatory target regions.

The difference in the potential scale of interactions, in terms of the number of interacting molecules during a response, between the central and peripheral proteins should be noted. In particular, the central proteins mainly correspond to the proteins involved in amplifying a light response, which may originate from as few as even a single rhodopsin molecule, while the peripheral proteins operate primarily at post-amplification quantities during signal transduction. In other words, these results indicate that the evolutionary constraint imposed on the topologically central proteins may derive not only from their higher number of interactions but also from the requirement that those interactions be reliable even at low levels of signal activation.

To date, several similar studies have been performed, relating molecular evolutionary histories with small-scale pathway structure (as opposed to interactome or metabolome-scale research). A correlation between topological measures and evolutionary rates was found in many cases even after the effect of other correlated factors, such as expression levels, had been ruled out. However, the specific pattern found varies between pathways. As many of these studies focused on metabolic pathways or signaling pathways with relatively linear structures, the results tend to focus on the relative evolutionary rates of upstream versus downstream proteins: in some pathways, the upstream proteins were found to be more constrained in their evolution [20,21,24,26-28], while in others it was the downstream proteins that were found to be under stronger constraint [22,23]. Still others have found branch points in the pathway to be an important location for adaptive evolution [25]. The structure of the phototransduction pathway, with its interplay between signal activation and parallel recovery and feedback mechanisms, prevents the simple assignment of such terms as “upstream” and “downstream”. Nevertheless, the results contained herein lend support to the body of evidence showing that a pathway’s topology influences the evolution of the proteins that comprise it, while a universal pattern of selective constraint imposed by the structure of a system is likely not to be found. Due to factors such as the aforementioned possible constraint imposed by low activated-molecule counts during early signal amplification events in the phototransduction response, the influence of system topology on molecular evolution should be considered on a per-pathway basis, in order to properly evaluate other system-derived constraints. To that extent, we have identified that there have been system-level patterns of natural selection acting on the primate phototransduction system.

## Methods

### Data set

A list of genes which encode for key proteins in the phototransduction cascade, the retinoid cycle and photoreceptor cell-type determination pathways in humans was culled from primary research articles and from literature reviews. The genes were subsequently categorized by three properties (Additional file 1: Table S1). The first was the process in which the proteins they encode are primarily active: photoreceptor cell-fate determination, phototransduction or the retinoid cycle. They were next determined to be either rod-specific, cone-specific or shared by both of these photoreceptor cell types. In the case of genes related to cell-fate determination, rod- or cone-specificity implies that the encoded protein functions to guide a cell exclusively towards a rod or a cone fate, respectively. Shared genes are those which are involved in general photoreceptor cell fate determination but are expressed before the explicit rod/cone decision has been made. For phototransduction genes, this categorization was determined by the cell type in which a given gene is expressed. Since the canonical retinoid cycle is utilized by both rods and cones, all genes categorized as belonging to this process were considered shared. Finally, the genes were classified by general function: retinoid cycle enzymes, chaperones, opsins, G proteins, phosphodiesterases, signal regulators, ion channels/exchangers, guanylate cyclases, or miscellaneous functions unique to individual proteins in the process.

The coding sequences of the human genes were downloaded along with the sequences of one-to-one orthologs from eight other mammalian species from the Ensembl database (release 60): *Pan troglodytes* (chimpanzee), *Gorilla gorilla* (gorilla), *Pongo pygmaeus* (orangutan), *Macaca mulatta* (macaque), *Callithrix jacchus* (marmoset), *Mus musculus* (mouse), *Rattus norvegicus* (rat), and *Canis familiaris* (dog). In the cases of multiple splice variants for a gene, the longest transcript was chosen. These species were chosen for the quality of their genomes and to provide sufficient divergence time for evolutionary rate analyses. Due to the current low quality of most mammalian genome databases, an analysis featuring a broader species representation would result in several genes being removed from the system-level analysis because of pervasive sequencing and assembly errors in some orthologs, which strongly influence evolutionary rate estimations.

Several orthologous sequences were either not present or found to be of poor quality in the Ensembl database so alternative sources were used when possible. The sequences of the rat ortholog of the gene *PRPH2* and the dog orthologs of *NR2E3* and *NEUROD1* were retrieved from NCBI GenBank (accession numbers X52376.1, XM_544754.2 and XM_545553.2, respectively). The sequence of the rat ortholog of the gene *NR2E3* was retrieved from the Rat Genome Database (RGD ID 2318602). The sequence of the rat ortholog of the gene *PDE6G* was retrieved from a primary literature source [41].

In three cases, genes were reported to have one-to-many orthologies. In the case of *SLC24A2*, two orthologous genes are annotated in the dog, ENSCAFG00000001623 and ENSCAFG00000023808. However, upon closer inspection and with the use of a BLAT search, which is a method similar to BLAST but designed to quickly find DNA sequences of high similarity, of the genome assembly using the human gene sequence as a query, it was determined that these two annotated sequences, in fact, together comprise the full orthologous gene and that the one-to-many orthology is a result of an annotation error that split the gene between the two entries. Thus, we reconstructed the full orthologous sequence from these two parts. The gene *PDE6C* has two orthologs annotated in the gorilla genome, ENSGGOG00000004416 and ENSGGOG00000024241. The latter of these two, however, is significantly shorter than the human gene and the other ortholog, so it was discarded. Finally, the gene *PDC* has two orthologs annotated in the gorilla genome, ENSGGOG00000002257 and ENSGGOG00000024688, which are highly similar to each other, however no full sequence gene duplication seems to have occurred and it is likely to be an artifact of the assembly process (Tomas Marquès-Bonet, personal communication) so the latter gene record was discarded.

When an orthologous sequence could not be retrieved from Ensembl or from alternative sources, it was predicted through bioinformatics means. First, a BLAT search was performed, using the sequence of the human genomic region containing the gene as a query against the target species’ genome assembly. For non-primate species, a second search using the sequence of the mouse genomic region containing the Murine orthologous gene would be used as a query, if possible, to complement the first search. In exceptional cases, a BLASTP search using the human and/or mouse amino acid sequence as a query was performed.

The gene *OPN1MW* (medium-wave/green cone opsin) was omitted from further analysis since its orthologous sequences could not be reliably retrieved for all species; aside from the human and orangutan, only one member of the tangential gene sequence consisting of *OPN1LW* and one or more copies of *OPN1MW* is present in each of the other Old World monkey genome assemblies. For example, the chimpanzee genome database only contains *OPN1LW* (ENSPTRG00000022427), while the gorilla database only contains *OPN1MW* (ENSGGOG00000004184). Furthermore, performing a similarity search via BLAT of either human gene against these assemblies only encounters one matching region. This suggests that, in these cases, the two highly similar genes were collapsed into a single one during the assembly process. While these genes have been independently sequenced for the chimpanzee, gorilla and orangutan [10], the complete nucleotide sequences are not publicly available.

Finally, in some cases in which a large portion of a sequence could not be recovered through bioinformatics means, we resequenced the DNA. Sequences from the Ensembl database were employed to design primers to be used in polymerase chain reactions (PCR). The following DNA samples from Barcelona Zoological Garden were used: PH19 (gorilla), PO3 (orangutan), PX11 (chimpanzee). For the genes *SLC24A1*, *PDE6B* and *CNGB1*, sequences from the human database were employed to design primers to be used in the polymerase chain reactions for the orangutan, gorilla and chimpanzee. For the gene *GRK1*, sequences from the macaque database were used to get sequences for the same species (orangutan, gorilla and chimpanzee). Mouse sequences were employed to design primers to amplify parts of *RGS9* in rat. All primers were 18-21 nucleotide-long. PCR reactions were carried out in a final volume of 50 *μ*l, including 1x activity buffer (EcoTaq DNA Polymerase.), 2 mM MgCl2, 200 *μ*M of each dNTP, 0.5 *μ*M primer, template DNA (approx. 30 to 40 ng), and 0.8 units of Taq polymerase (EcoTaq DNA Polymerase). Amplifications were run in a Gene Amp PCR System 9700 (Applied Biosystem) thermocycler programmed as follows: a preliminary 5-min. denaturation at 94°C; 30 cycles of 30 sec at 94°C (denaturation), 1 min. at specific PCR annealing temperatures, and 1.5 min. at 72°C (extension); and a final extension at 72°C for 5 min. followed by storage at 4°C. Because of failures in the amplifications, additional primers were used in some cases. Electrophoresis was performed in 1.4% agarose gels (Sigma). These amplified products were gel purified and directly sequenced on both strands. Fragments amplified with the most external primers were sequenced with the appropriate internal primers. All sequences are available on GenBank (See Additional file 1: Table S4 for accession numbers).

The multiple alignments of the CDS of the orthologous sequences as computed by the GeneTrees method [42] were downloaded from the Ensembl Compara database (release 60) via the Perl interface. In cases in which sequences were retrieved from alternate sources, were supplemented by BLAT searches or for which the DNA was resequenced, multiple alignments of the corresponding amino acid sequences were created using *TCoffee* version 8.13 [43] and then back-translated to nucleotide sequence alignments. Next, poorly aligned regions were automatically removed by codons from each alignment using *GBlocks* version 0.91b [44]. Finally, the alignments were visually inspected for further refinement. It should be noted that GBlocks is very conservative in its filtering, as has been demonstrated recently, and its aggressive elimination of poorly aligned regions may result in the under-identification of positively selected sites [45]. On the other hand, the stringent process that we employed also provided higher confidence that any detected event of positive selection is true.

### Phototransduction network

To gain an understanding of the interactions between the proteins comprising the phototransduction pathway, we represented the system as a network, in which nodes represented the proteins and edges represented the interactions between them. In defining interactions, we included both the formation of stable complexes and transient interactions. A list of 81 protein-protein interactions was curated from both primary research articles and literature reviews (Additional file 1: Table S2). Thus, all interactions in the network are supported by experimental evidence, however it should be noted that some interactions have only been described in single experiments while others are well understood and widely supported. Also noteworthy is that the network was built with the assumption that the structure of the pathway is conserved between mammalian species. One known exception to this is that rhodopsin kinase (*GRK1*), which is expressed in both rods and cones in rodents and primates and is presumed to interact with both rhodopsin and cone opsins in these species, is not expressed in canine cone cells [46]. As the network was largely constructed from knowledge of human and mouse physiology, we chose to retain for this protein both its rod- and cone-specific interactions.

Basic node properties were computed for each gene of the phototransduction network. One method for describing the position of proteins within a network is to measure their centrality. There are several different measurements of centrality in the study of networks, which may be interpreted differently in a biological context. We considered two different measurements of centrality in this study: betweenness centrality, a measurement related to the number of shortest paths between proteins in the network that go through a given protein, the value of which, when high, indicates that the protein is a sort of information “bridge” in the system; and closeness centrality, a measurement related to the average distance of a protein to all the other proteins in the network, which may be interpreted as being the closest to our common-language understanding of the word “centrality”. Finally, we also considered the degree, or connectivity, of the proteins, which may be an indicator of importance in the system. All network properties were computed using the Python library *NetworkX* version 1.2 [47]. A graphical representation of the network was created using *Cytoscape* version 2.8.3 [48] (Figure 1).

### Estimates of evolutionary rates

The rate of evolution for each gene was estimated as *ω*, or the ratio of the non-synonymous to the synonymous rates of substitution (*d*_*N*_/*d*_*S*_), as computed in the M0 model of the program *codeml* from the PAML package version 4.4c [29]. The rates were estimated for the divergence of the entire phylogenetic tree. These are usually expected to be less than one due to a predominantly purifying selective force maintaining overall protein structure and function over time. Estimates were made for each gene using both the species tree and a gene tree computed using *PhyML* with the default parameters [49]. The tree which resulted in the M0 model fitting the data with the higher maximum likelihood was chosen for further analyses.

All models (including those used in tests of positive selection; see below) were estimated using three different starting *ω* values (0.1, 1.0, and 2.0) to account for local maxima. Each combination of parameters was modeled five times to check for consistency of the results.

### Tests of positive selection

Each gene was tested for evidence of positive selection events using *codeml*. To test for evidence of positive selection using the entire tree (selected while calculating the rates of evolution), two likelihood ratio tests were performed, comparing model M2a to model M1a and comparing model M8 to model M7. The former comparison tends to be more robust, while the latter tends to be more sensitive [50]. Both of these tests compare nested models in which the alignment is partitioned into sites and one of the two models explicitly allows some sites to be under positive selection (*ω*>1) (M2a and M8) and the other restricts all sites to either purifying selection (*ω*<1) or neutral evolution (*ω*=1) (the null models M1a and M7). Both comparisons were tested for significance via a likelihood ratio test with 2 degrees of freedom and *p*-values were corrected for multiple testing against tests performed for all the genes [51].

In order to test whether any gene experienced events of positive selection along individual internal branches of the species tree, tests of positive selection were made using branch-site model A of *codeml*[34]. With branch-site model A, one branch of the tree is designated as the “foreground” and the rest of the tree is the “background”. Next the multiple sequence alignment is partitioned into sites which are assigned one of four classes: class 0, in which both the background and foreground are under purifying selection (0<*ω*_0_<1); class 1, in which both the background and foreground are under neutral evolution (*ω*_1_=1); class 2a, in which the background is under purifying selection (0<*ω*_0_<1) and the foreground is under positive selection (*ω*_2_≥1); and class 2b, in which the background is under neutral evolution (*ω*_1_=1) and the foreground is under positive selection (*ω*_2_≥1). The model was tested for significance by a likelihood ratio test with a null model which fixes *ω*_2_=1, with 1 degree of freedom and *p*-values were corrected for multipule testing against tests performed for all the genes across all branches [51].

Finally, in cases in which positive selection events were detected by these tests, the amino acids most likely to have been under positive selection were identified by the Bayes Empirical Bayes method as implemented in *codeml*[52]. The Bayes Empirical Bayes method assigns posterior probabilities that a codon site evolves at *ω*>1 through a Bayesian method which accounts for uncertainties in the model parameters.

### Statistical analyses

The relationships of protein length and network parameters with the substitution rates (*ω*, *d*_*N*_, and *d*_*S*_) were tested by computing Spearman’s *ρ* correlation coefficient. The effects of the gene classifications (process, photoreceptor cell-type specificity and function) on all estimated rates were tested using the Kruskal-Wallis rank sum test. Within the network, correlations between estimated rates for each node and the mean estimated rates for their immediate neighbors were estimated using Spearman’s *ρ*. All statistical computations were performed using R [53].

## Competing interests

The authors declare that they have no competing interests.

## Authors’ contributions

BI constructed the phototransduction network, assisted in the implementation of the bioinformatic pipeline, performed the statistical analyses and drafted the manuscript. LM designed and implemented the bioinformatic pipeline. HL coordinated the DNA resequencing and assisted with the statistical analyses. JB conceived of and coordinated the study. All authors read and approved the final manuscript.

## Supplementary Material

Additional file 1 **Supplementary tables.** Contains three, multi-page supplementary tables, Tables S1, S2 and S3. Click here for file
