# Supplementary material for: A system-level, molecular evolutionary analysis of mammalian phototransduction
Source: BMC Evol Biol. 2013 Feb 23;13:52. doi: 10.1186/1471-2148-13-52 (PMC3616935; doi:10.1186/1471-2148-13-52)
Supplement: Additional file 1 — Supplementary tables. Contains three, multi-page supplementary tables, Tables S1, S2 and S3. [file 1471-2148-13-52-S1.pdf]

A system-level, molecular evolutionary analysis of mammalian phototransduction (supplementary material)

Brandon M Invergo[1] , Ludovica Montanucci*[1] , Hafid Laayouni[1] and Jaume Bertranpetit[1]

[1]IBE-Institute of Evolutionary Biology (UPF-CSIC), CEXS-UPF-PRBB, Barcelona, Catalonia, Spain

Email: Brandon Invergo - brandon.invergo@upf.edu; Ludovica Montanucci*- ludovica.montanucci@upf.edu; Hafid Laayouni - hafid.laayouni@upf.edu; Jaume Bertranpetit - jaume.bertranpetit@upf.edu;

*Corresponding author

Table S1 - Classifications of the genes

Genes were assigned classifications according to their photoreceptor cell-type specificity, the process in which the encoded protein is primarily active, and the general function of the encoded protein. (Note: here "enzyme" specifically refers to enzymes involved in retinoid recycling.)

gene	cell type	process	function
ABCA4	shared	retinoid cycle	enzyme
AIPL1	shared	phototransduction	other
ARR3	cone	phototransduction	signal regulator
ASCL1	rod	development	transcription regulation
CNGA1	rod	phototransduction	ion channel
CNGA3	cone	phototransduction	ion channel
CNGB1	rod	phototransduction	ion channel
CNGB3	cone	phototransduction	ion channel
CRX	shared	development	transcription regulation
GNAT1	rod	phototransduction	G protein
GNAT2	cone	phototransduction	G protein
GNB1	rod	phototransduction	G protein
GNB3	cone	phototransduction	G protein
GNB5	shared	phototransduction	G protein
GNGT1	rod	phototransduction	G protein
GNGT2	cone	phototransduction	G protein
GPSM2	shared	phototransduction	other
GRK1	shared	phototransduction	signal regulator
GUCA1A	shared	phototransduction	signal regulator
GUCA1B	shared	phototransduction	signal regulator
GUCY2D	shared	phototransduction	other
GUCY2F	shared	phototransduction	other
LRAT	shared	retinoid cycle	enzyme
NEUROD1	shared	development	transcription regulation
NR2E3	rod	development	transcription regulation
NR2F1	cone	development	transcription regulation
NRL	rod	development	transcription regulation
OPN1SW	cone	phototransduction	other
OTX2	shared	development	transcription regulation
PDC	shared	phototransduction	signal regulator
PDE6A	rod	phototransduction	phosphodiesterase
PDE6B	rod	phototransduction	phosphodiesterase
PDE6C	cone	phototransduction	phosphodiesterase
PDE6G	rod	phototransduction	phosphodiesterase
PDE6H	cone	phototransduction	phosphodiesterase
PIAS3	rod	development	transcription regulation
PRPH2	shared	development	other
RBP1	shared	retinoid cycle	chaperon
RBP3	shared	retinoid cycle	chaperon

gene	cell type	process	function
RCVRN	shared	phototransduction	signal regulator
RDH12	shared	retinoid cycle	enzyme
RDH5	shared	retinoid cycle	enzyme
RDH8	shared	retinoid cycle	enzyme
RGR	shared	retinoid cycle	enzyme
RGS9	shared	phototransduction	signal regulator
RGS9BP	shared	phototransduction	other
RHO	rod	phototransduction	other
RLBP1	shared	retinoid cycle	chaperon
ROM1	shared	development	other
RORA	cone	development	transcription regulation
RORB	shared	development	transcription regulation
RPE65	shared	retinoid cycle	enzyme
RXRG	cone	development	transcription regulation
SAG	rod	phototransduction	signal regulator
SLC24A1	rod	phototransduction	ion channel
SLC24A2	cone	phototransduction	ion channel
THRB	cone	development	transcription regulation

Table S2 - Protein interactions in the phototransduction signaling pathway

The status of each interaction is defined as "confirmed", or generally considered to be true by multiple sources such as reviews; "suggested", or proposed by a primary research source but not replicated; or "inferred", or inferred from a primary research source, such as indirect interactions across a molecular complex where no direct interaction is described or interactions of a paralogous protein to that described in the research.

Gene 1	Gene 2	Status	Primary Source
SAG	SAG	confirmed	[1]
GRK1	RCVRN	confirmed	[2]
SLC24A2	CNGA3	confirmed	[3]
SLC24A2	CNGB3	confirmed	[3]
SLC24A1	CNGA1	confirmed	[3]
SLC24A1	CNGB1	confirmed	[3]
CNGB1	PRPH2	confirmed	[4]
CNGB3	CNGA3	confirmed	[5]
CNGA3	CNGA3	confirmed	[5]
CNGB3	CNGB3	confirmed	[5]
CNGA1	CNGB1	confirmed	[6]
CNGA1	CNGA1	confirmed	[7]
CNGB3	PDE6C	inferred	[8]
CNGB3	PDE6H	inferred	[8]
CNGB1	PDE6A	suggested	[8]
CNGB1	PDE6B	suggested	[8]
CNGB1	PDE6G	suggested	[8]
GPSM2	GNAT2	suggested	[9]
GPSM2	GNAT1	suggested	[9]
PDE6C	GNAT2	inferred	[10]
PDE6H	GNAT2	inferred	[11]
GNAT1	PDE6A	inferred	[10]
GNAT1	PDE6G	confirmed	[11]
GNAT1	PDE6B	inferred	[10]
ARR3	OPN1SW	confirmed	[12]
GRK1	OPN1SW	confirmed	[12]
OPN1SW	GNAT2	inferred	[13, 14]
OPN1SW	GNB3	inferred	[15]
OPN1SW	GNGT2	inferred	[16]
SAG	RHO	confirmed	[17]
GRK1	RHO	confirmed	[18]
RHO	GNAT1	confirmed	[19]
RHO	GNB1	confirmed	[19]
RHO	GNGT1	confirmed	[19]
PRPH2	PRPH2	confirmed	[20]
PRPH2	ROM1	confirmed	[20]
PDC	GNB3	confirmed	[21]
PDC	GNGT2	confirmed	[21]
PDC	GNB1	confirmed	[22]

Gene 1	Gene 2	Status	Source
PDC	GNGT1	confirmed	[22]
PDE6C	PDE6H	confirmed	[23, 24]
PDE6C	PDE6C	inferred	[24]
PDE6H	PDE6H	inferred	[25]
PDE6A	PDE6G	confirmed	[26]
PDE6A	PDE6B	confirmed	[26]
PDE6B	PDE6G	confirmed	[26]
PDE6G	PDE6G	inferred	[25]
GUCY2D	GUCY2F	inferred	[27]
GUCY2D	GUCY2D	confirmed	[27]
GUCY2F	GUCY2F	inferred	[27]
GNAT2	GUCY2D	confirmed	[28]
GNAT2	GUCY2F	confirmed	[28]
GNAT1	GUCY2D	confirmed	[28]
GNAT1	GUCY2F	confirmed	[28]
GUCA1A	GUCY2F	confirmed	[29]
GUCA1B	GUCY2F	confirmed	[29]
GUCA1A	GUCY2D	confirmed	[29]
GUCA1B	GUCY2D	confirmed	[29]
RGS9	GUCY2F	confirmed	[30, 31]
RGS9	GUCY2D	confirmed	[30, 31]
RGS9	RGS9BP	confirmed	[32]
GNB5	RGS9	confirmed	[33]
GNB5	GNAT1	inferred	[34]
RGS9	PDE6G	confirmed	[34, 35]
RGS9	GNAT1	confirmed	[34]
GNAT2	RGS9	inferred	[34]
GNB5	GNAT2	inferred	[34]
PDE6H	RGS9	inferred	[34, 35]
ROM1	ROM1	confirmed	[20]
GNAT2	GNB3	inferred	[14, 15, 19]
GNAT2	GNGT2	inferred	[14, 16, 19]
GNB3	GNGT2	inferred	[15, 16, 19]
GNAT1	GNB1	confirmed	[19]
GNAT1	GNGT1	confirmed	[19]
GNB1	GNGT1	confirmed	[19]

Table S3 - Results

The results calculated for each gene are listed: the human protein length in amino acids, the non-synonymous substitution rate (d_N), the synonymous substitution rate (d_S), and the ratio d_N/d_S (ω).

gene	length	d_N	d_S	ω
ABCA4	2273	0.13	0.94	0.139
AIPL1	384	0.095	2.329	0.041
ARR3	388	0.178	0.802	0.222
ASCL1	236	0.012	1.534	0.008
CNGA1	759	0.096	1.043	0.092
CNGA3	698	0.156	1.452	0.107
CNGB1	1251	0.173	1.423	0.121
CNGB3	809	0.253	0.81	0.312
CRX	299	0.052	1.322	0.039
GNAT1	350	0.004	1.894	0.002
GNAT2	354	0.041	0.882	0.047
GNB1	340	0.003	1.047	0.003
GNB3	340	0.042	1.094	0.039
GNB5	395	0.011	1.011	0.01
GNGT1	74	0.037	1.01	0.037
GNGT2	69	0.119	1.056	0.113
GPSM2	684	0.076	0.994	0.077
GRK1	563	0.157	2.422	0.065
GUCA1A	205	0.052	2.569	0.02
GUCA1B	200	0.056	1.542	0.036
GUCY2D	1103	0.158	1.533	0.103
GUCY2F	1108	0.122	0.621	0.196
LRAT	230	0.215	1.367	0.157
NEUROD1	356	0.037	1.407	0.026
NR2E3	410	0.079	1.386	0.057
NR2F1	423	0.004	0.927	0.004
NRL	237	0.191	1.165	0.164
OPN1SW	348	0.157	0.972	0.162
OTX2	297	0.023	0.377	0.06
PDC	270	0.099	0.918	0.108
PDE6A	860	0.055	1.187	0.046
PDE6B	854	0.1	1.884	0.053
PDE6C	858	0.142	1.051	0.135
PDE6G	137	0.022	0.817	0.027
PDE6H	83	0.091	0.953	0.096
PIAS3	628	0.042	0.771	0.054
PRPH2	346	0.08	1.579	0.051
RBP1	197	0.023	1.141	0.021
RBP3	1274	0.161	2.025	0.08

gene	length	d_N	d_S	ω
RCVRN	200	0.121	1.293	0.094
RDH12	316	0.125	1.104	0.113
RDH5	318	0.129	0.883	0.146
RDH8	311	0.149	1.183	0.126
RGR	297	0.219	1.556	0.141
RGS9	674	0.117	1.275	0.092
RGS9BP	235	0.179	2.468	0.073
RHO	348	0.052	1.905	0.027
RLBP1	347	0.091	1.461	0.063
ROM1	198	0.209	0.794	0.263
RORA	556	0.012	0.697	0.017
RORB	470	0.025	0.574	0.043
RPE65	533	0.061	0.98	0.062
RXRG	463	0.027	1.035	0.027
SAG	405	0.137	1.09	0.126
SLC24A1	1099	0.4	0.91	0.44
SLC24A2	661	0.107	0.696	0.153
THRB	476	0.041	0.668	0.062

Table S4 - GenBank Accession Numbers for resequenced exons

Several exons were resequenced to account for their absence or poor sequence quality in the public genome databases. All exon sequences are available on GenBank

gene	species	exon	Accession Number
CNGB1	*P. pygmaeus*	1	KC484731
CNGB1	*P. pygmaeus*	24	KC484732
CNGB1	*P. troglodytes*	5	KC484733
CNGB1	*P. troglodytes*	6	KC484734
CNGB1	*P. troglodytes*	24	KC484735
CNGB1	*G. gorilla*	28	KC484736
GRK1	*P. troglodytes*	3	KC484737
PDE6B	*P. troglodytes*	2	KC484738
PDE6B	*P. troglodytes*	4	KC484739
PDE6B	*P. troglodytes*	9	KC484740
PDE6B	*P. troglodytes*	10	KC484741
PDE6B	*P. troglodytes*	13	KC484742
PDE6B	*P. troglodytes*	14	KC484743
PDE6B	*P. troglodytes*	21	KC484744
PDE6B	*P. pygmaeus*	5	KC484745
PDE6B	*P. pygmaeus*	10	KC484746
RGS9	*R. norvegicus*	1	KC484747
RGS9	*R. norvegicus*	6	KC484748
SLC24A1	*P. pygmaeus*	7	KC484749
SLC24A1	*G. gorilla*	7	KC484750

References

1. Hanson SM, Van Eps N, Francis DJ, Altenbach C, Vishnivetskiy SA, Arshavsky VY, Klug CS, Hubbell WL, Gurevich VV: **Structure and function of the visual arrestin oligomer**. *The EMBO Journal* 2007, **26**(6):1726–1736, [http://www.ncbi.nlm.nih.gov/pubmed/17332750].

2. Chen CK, Inglese J, Lefkowitz RJ, Hurley JB: **Ca(2+)-dependent interaction of recoverin with rhodopsin kinase.** *The Journal of Biological Chemistry* 1995, **270**(30):18060–18066.

3. Kang K, Bauer PJ, Kinjo TG, Szerencsei RT, Bönigk W, Winkfein RJ, Schnetkamp PPM: **Assembly of retinal rod or cone Na(+)/Ca(2+)-K(+) exchanger oligomers with cGMP-gated channel subunits as probed with heterologously expressed cDNAs**. *Biochemistry* 2003, **42**(15):4593–4600, [http://www.ncbi.nlm.nih.gov/pubmed/12693957].

4. Poetsch A, Molday LL, Molday RS: **The cGMP-gated channel and related glutamic acid-rich proteins interact with peripherin-2 at the rim region of rod photoreceptor disc membranes**. *Journal of Biological Chemistry* 2001, **276**(51):48009–48016.

5. Peng C, Rich ED, Varnum MD: **Subunit Configuration of Heteromeric Cone Cyclic Nucleotide-Gated Channels**. *Neuron* 2004, **42**(3):401–410.

6. Chen T, Illing M, Molday L, Hsu Y, Yau K, Molday R: **Subunit 2 (or beta) of retinal rod cGMP-gated cation channel is a component of the 240-kDa channel-associated protein and mediates Ca(2+)-calmodulin modulation.** *Proceedings of the National Academy of Sciences* 1994, **91**(24):11757–11761.

7. Zheng J, Trudeau MC, Zagotta WN: **Rod Cyclic Nucleotide-Gated Channels Have a Stoichiometry of Three CNGA1 Subunits and One CNGB1 Subunit**. *Neuron* 2002, **36**(5):891–896.

8. Körschen HG, Beyermann M, Müller F, Heck M, Vantler M, Koch KW, Kellner R, Wolfrum U, Bode C, Hofmann KP, Kaupp UB: **Interaction of glutamic-acid-rich proteins with the cGMP signalling pathway in rod photoreceptors**. *Nature* 1999, **400**(6746):761–766, [http://www.ncbi.nlm.nih.gov/pubmed/10466724].

9. Kerov VS, Natochin M, Artemyev NO: **Interaction of transducin-α with LGN, a G-protein modulator expressed in photoreceptor cells**. *Molecular and Cellular Neuroscience* 2005, **28**:485–495.

10. Guo L, Hajipour A, Ruoho A: **Complementary interactions of the rod PDE6 inhibitory subunit with the catalytic subunits and transducin**. *Journal of Biological Chemistry* 2010, **285**(20):15209–15219.

11. Antonny B, Otto-Bruc A, Chabre M, Vuong T: **GTP hydrolysis by purified alpha-subunit of transducin and its complex with the cyclic GMP phosphodiesterase inhibitor**. *Biochemistry* 1993, **32**(33):8646–8653.

12. Zhu X, Brown B, Li A, Mears A, Swaroop A, Craft C: **GRK1-dependent phosphorylation of S and M opsins and their binding to cone arrestin during cone phototransduction in the mouse retina**. *Journal of Neuroscience* 2003, **23**(14):6152–6160.

13. Lerea C, Bunt-Milam A, Hurley J: **Alpha transducin is present in blue-, green-, and red-sensitive cone photoreceptors in the human retina**. *Neuron* 1989, **3**(3):367–376.

14. Lerea C, Somers D, Hurley J, Klock I, Bunt-Milam A: **Identification of specific transducin alpha subunits in retinal rod and cone photoreceptors**. *Science* 1986, **234**(4772):77–80.

15. Lee R, Lieberman B, Yamane H, Bok D, Fung B: **A third form of the G protein beta subunit. 1. Immunochemical identification and localization to cone photoreceptors**. *Journal of Biological Chemistry* 1992, **267**(34):24776–24781.

16. Ong O, Yamane H, Phan K, Fong H, Bok D, Lee R, Fung B: **Molecular cloning and characterization of the G protein gamma subunit of cone photoreceptors**. *Journal of Biological Chemistry* 1995, **270**(15):8495–8500.

17. Pfister C, Chabre M, Plouet J, Tuyen V, De Kozak Y, Faure J, Kühn H: **Retinal S antigen identified as the 48K protein regulating light-dependent phosphodiesterase in rods**. *Science* 1985, **228**(4701):891–893.

18. Palczewski K, McDowell J, Hargrave P: **Rhodopsin kinase: substrate specificity and factors that influence activity**. *Biochemistry* 1988, **27**:2306–2313.

19. Fung B, Hurley J, Stryer L: **Flow of information in the light-triggered cyclic nucleotide cascade of vision**. *Proceedings of the National Academy of Sciences* 1981, **78**:152–156.

20. Moritz OL, Molday RS: **Molecular cloning, membrane topology, and localization of bovine rom-1 in rod and cone photoreceptor cells**. *Investigative Ophthalmology & Visual Science* 1996, **37**(2):352–362, [http://www.ncbi.nlm.nih.gov/pubmed/8603840].

21. Chen F, Ng PS, Faull KF, Lee RH: **Cone photoreceptor $\beta\gamma$-transducin: posttranslational modification and interaction with phosducin**. *Investigative Ophthalmology & Visual Science* 2003, **44**(11):4622–4629, [http://www.iovs.org/cgi/doi/10.1167/iovs.03-0420].

22. Kuo C, Taniura H, Watanabe Y, Fukada Y, Yoshizawa T, Miki N: **Identification of a retina-specific MEKA protein as a 33 K protein**. *Biochemical and Biophysical Research Communications* 1989, **162**(3):1063–1068.

23. Gillespie P, Beavo J: **Characterization of a bovine cone photoreceptor phosphodiesterase purified by cyclic GMP-sepharose chromatography**. *The Journal of Biological Chemistry* 1988, **263**(17):8133–8141.

24. Hurwitz R, Bunt-Milam A, Chang M, Beavo J: **cGMP phosphodiesterase in rod and cone outer segments of the retina**. *The Journal of Biological Chemistry* 1985, **260**:568–573.

25. Deterre P, Bigay J, Forquet F, Robert M, Chabre M: **cGMP phosphodiesterase of retinal rods is regulated by two inhibitory subunits.** *Proceedings of the National Academy of Sciences* 1988, **85**(8):2424–2428.

26. Hurley J, Stryer L: **Purification and characterization of the gamma regulatory subunit of the cyclic GMP phosphodiesterase from retinal rod outer segments**. *The Journal of Biological Chemistry* 1982, **257**(18):11094–11099.

27. Yu H, Olshevskaya E, Duda T, Seno K, Hayashi F, Sharma R, Dizhoor A, Yamazaki A: **Activation of retinal guanylyl cyclase-1 by Ca2+-binding proteins involves its dimerization**. *The Journal of Biological Chemistry* 1999, **274**:15547–15555.

28. Rosenzweig DH, Nair KS, Levay K, Peshenko IV, Crabb JW, Dizhoor AM, Slepak VZ: **Interaction of retinal guanylate cyclase with the α subunit of transducin: potential role in transducin localization**. *Biochemical Journal* 2009, **417**:803–812.

29. Koch KW, Duda T, Sharma RK: **Photoreceptor specific guanylate cyclases in vertebrate phototransduction**. *Molecular and Cellular Biochemistry* 2002, **230**:97–106.

30. Yu H, Bondarenko VA, Yamazaki A: **Inhibition of retinal guanylyl cyclase by the RGS9-1 N-terminus**. *Biochemical and Biophysical Research Communications* 2001, **286**:12–19, [http://www.ncbi.nlm.nih.gov/pubmed/11485301].

31. Seno K, Kishigami A, Ihara S, Maeda T, Bondarenko V, Nishizawa Y, Usukura J, Yamazaki A, Hayashi F: **A possible role of RGS9 in phototransduction. A bridge between the cGMP-phosphodiesterase system and the guanylyl cyclase system**. *The Journal of Biological Chemistry* 1998, **273**(35):22169–22172.

32. Hu G, Wensel T: **R9AP, a membrane anchor for the photoreceptor GTPase accelerating protein, RGS9-1**. *Proceedings of the National Academy of Sciences* 2002, **99**(15):9755–9760.

33. Makino E, Handy J, Li T, Arshavsky V: **The GTPase activating factor for transducin in rod photoreceptors is the complex between RGS9 and type 5 G protein beta subunit**. *Proceedings of the National Academy of Sciences* 1999, **96**(5):1947–1952.

34. He W, Cowan C, Wensel T: **RGS9, a GTPase accelerator for phototransduction**. *Neuron* 1998, **20**:95–102.

35. Slep KC, Kercher MA, He W, Cowan CW, Wensel TG, Sigler PB: **Structural determinants for regulation of phosphodiesterase by a G protein at 2.0Å**. *Nature* 2001, **409**(6823):1071–1077.
